# Acute and Subacute Toxicity Studies of the Aqueous Extract from *Haloxylon scoparium* Pomel (*Hammada scoparia* (Pomel)) by Oral Administration in Rodents

**DOI:** 10.1155/2020/4020647

**Published:** 2020-10-06

**Authors:** Loubna Kharchoufa, Mohamed Bouhrim, Noureddine Bencheikh, Soufiane El Assri, Asmae Amirou, Amal Yamani, Mohammed Choukri, Hassane Mekhfi, Mostafa Elachouri

**Affiliations:** ^1^Laboratory of Bioresources, Biotechnology, Ethnopharmacology and Health, URAC-40, Department of Biology, Faculty of Sciences, Mohammed First University, Oujda, Morocco; ^2^Biochemistry Laboratory, Central Laboratory Service-CHU, Mohammed VI, Oujda, Morocco; ^3^Faculty of Medicine and Pharmacy, Oujda, Morocco

## Abstract

**Materials and Methods:**

Acute toxicity test was performed on Swiss albino mice at a single oral dose of 1-10 g/kg for 14 consecutive days. General behavioral adverse effects, mortality, and latency of mortality were determined. In the subacute study, the *Haloxylon scoparium* Pomel extract was administered orally at doses of 500, 1000, and 2000 mg/kg daily for 30 days to Wistar rats. Body weight and selected biochemical and hematological parameters were determined at the end of the experiment. Sections of livers and kidneys were removed for histological studies.

**Results:**

Acute toxicity study showed that the oral LD_50_ value of *Haloxylon scoparium* Pomel extract was 5000 mg/kg. The subacute toxicity study of *Haloxylon scoparium* Pomel extract at doses 500, 1000, and 2000 mg/kg did not produce any observable symptoms of toxicity and no significant variation in body weight, organ weights, food, and water consumption or mortality in all treated rats. However, the administration of the *Haloxylon scoparium* Pomel extract to rats at 500 mg/kg and 1000 mg/kg showed a significant decrease in platelets. Moreover, only at the highest dose (2000 mg/kg), the extract caused a significant increase in red blood cells and hemoglobin. Our results showed that subacute treatments with *Haloxylon scoparium* Pomel extract at doses of 1000 mg/kg and 2000 mg/kg significantly elevated alkaline phosphatase and triglycerides. Histological studies showed that the subacute treatments of rats with *Haloxylon scoparium* Pomel extracts, at the doses 1000 and 2000 mg/kg, induced some histopathological changes in the livers but a slight changing in kidneys.

**Conclusion:**

Our results indicated low acute toxicity of the aqueous extract of *Haloxylon scoparium* Pomel. Furthermore, daily oral administration of *Haloxylon scoparium* Pomel extract caused some damages to the livers of rats treated with high doses, expressed by an increase in some enzyme activities such as ALP. Regarding the renal function, we did not find remarkable toxicity in the subacute treatment with *Haloxylon scoparium* Pomel extracts at doses 1000 and 2000 mg/kg. However, further toxicity assessments should be done to ascertain the safety or the toxicity of this valuable plant species “*Haloxylon scoparium* pomel” in subchronic treatments.

## 1. Introduction

Since ancient times, herbs were widely used as the main treatment strategy for treating diseases [1]. Currently, this botanical medicine is increasingly becoming popular throughout the world, especially in developing countries, where medicinal plants are available, accessible, and are at the reach of the poor people. Even though the use of these plants has shown promising potential phytotherapeutic effects with high global demand, but there are still concerns about not only their use but also their safety [2]. In fact, in Moroccan society, based on their long history of uses, medicinal plants are usually regarded as safe or of low toxicity [3–6]. In this country, users believe that medicinal plants have fewer side effects than synthetic drugs. However, the general perception that herbal drugs are very safe and devoid of adverse effects is not entirely true, but misleading, taking in mind, numerous surveys, conducted by our team, confirming the toxicity of medicinal plants used by Moroccan people [7–9]. Additionally, previous reports issuing from the Moroccan Poison Center “MPC,” indicated that herbs are the cause of 3–5% of all reported intoxications, of which 17% were associated with fatal events [10]. Indeed, the safety of these wealthy herbs continues to be a major issue with their uses. So, the importance of such investigation is needed to ascertain the safety profile of valuable plant species commonly used traditionally by Moroccan society.

*Haloxylon scoparium* or *Hammada scoparia* (Pomel) belongs to the Amaranthaceae family and is locally known as “*Rremt*” in Morocco. It is a common herb amongst practitioners, herbalists, and users. This popular plant is widely used as a decoction, infusion, or cataplasm to treat various ailments. It has frequently used in the treatment of hypertension, cutaneous neoplasms, dermatitis, diabetes, food poisoning, rheumatoid arthritis, osteoarthritis, scabies, injury healing, indigestion, stomachache, gastroenteritis, and cold [6, 11–13]. Moreover, the plant is also used as an antidote of scorpion stings and snakebites [11]. The leaves, infused or decocted, are used as a mouthwash to treat mouth diseases and toothache [14]. Pharmacological studies have shown that the plant has antimicrobial, antioxidant activities, larvicidal activity, cytotoxic and antimalarial activities, molluscicidal activity, anticancer properties, reno-protective, and hepatoprotective effects. Furthermore, a recent study suggested that HS extract could possibly restore the altered neurological capacities and antioxidant power in rats [15]. Regarding the chemical composition, two alkaloids (N-methylisosalsoline and carnegine) had been previously described from the aerial parts of *Hammada articulata* ssp. scoparia. A thorough study of this plant material has now led to the isolation of eight minor alkaloids and one flavonoid. Several alkaloids, including four isoquinolines (isosalsoline, salsolidine, dehydrosalsolidine, and isosalsolidine), one isoquinolone (N-methylcorydaldine), tryptamine, N-omega-methyltryptamine, and one beta-carboline (tetrahydroharman), have been extracted for this plant [16]. Also, some flavonoids have been identified as isorhamnetin-3-O-beta-D-robinobioside [16]. Another analysis of the flavonoid-enriched extract from HS by LC-UV-MS/MS indicated the presence of isorhamnetin–xylose–galactose, quercetin–xylose–rhamnose–galactose, and quercetin–glucose–rhamnose (rutin) [17].

Despite the great phytotherapeutic importance of HS, the assessment of the safety or toxicity of this species has completely neglected; yet, it was cited, in a recent ethnobotanical investigation conducted by our team, as toxic [8]. In this respect, we undertook this work to assess the possible acute and subacute toxic effects of the aqueous extract of this plant.

## 2. Materials and Methods

### 2.1. Plant Material

The aerial parts of HS was collected in April 2019 at flowering stage in a desert area situated between Tendrara and Figuig (South-Eastern Morocco), exactly at the district named Abbou Lakhal. The plant was identified by Professor Mohammed Fennane from the Scientific Institut of Rabat. A voucher specimen of HS was deposited at the Herbarium Mohammed First University, Oujda, Morocco (HUMPOM451). The name of the plant was checked and confirmed according to the official website (https://www.theplantlist.org).

### 2.2. Preparation of the Aqueous Extract of *Haloxylon scoparium* Pomel

Aerial parts of HS were air-dried at room temperature for 15 days and then used for toxicities studies. The powdered aerial parts of HS (80 g) were extracted with boiling water (800 ml) for 20 min, after which the boiled decoction was filtered and then concentrated in a rotary vacuum evaporator. The yield extract was about 21.25%. The extracted material was stored at −20°C in the dark until further use.

### 2.3. Experimental Animals

Wistar rats and Swiss albino mice were provided from the local colonies of the department of biology (Faculty of Sciences-Oujda, Morocco), they were placed under standard conditions (23°C ± 2°C and 12 h light-dark cycle), and they were allowed to free access of water and food. All animals were cared for in compliance with the internationally accepted guide for the care and use of laboratory animals, published by the US National Institutes of Health (NIH Publication No. 85-23, Revised in 1985).

### 2.4. Acute Toxicity Studies in Mice

The single-dose acute oral toxicity study was evaluated following the recommendations by OECD Guidelines (425) [18]. Acute toxicity studies were carried out in Swiss albino mice, weighing 20–30 g each one, using a single dose, which administered orally. Thirty-six mice, divided into 6 groups, were designed for the study of acute toxicity via the oral route. Each group of 6 mice (3 males and 3 females) received, respectively, a single oral dose of 1, 3, 5, 7, and 10 g/kg body weight of HS extract, while the control group was treated with distilled water. The general behavior of mice and signs of toxicity were observed continuously for 1 h after the oral treatment and then intermittently for 4 h and thereafter over a period of 24 h [19]. The mice were further observed once a day up to 14 days for following treatment for behavioral changes and signs of toxicity and/or death and the latency of death. The LD_50_ value was determined according to Dragstedt and Lang method described by El Allaoui [20].

### 2.5. Subacute Toxicity

#### 2.5.1. Treatments

For the study of subacute toxicity, four experimental groups were established (6 animals/group; 3 males and 3 females), as follows: control group (treated orally with distilled water for a period of 30 days) and test groups (treated by gavage for 30 days with different doses of the aqueous extract: 500, 1000, or 2000 mg/kg body weight). The doses for the subacute toxicity test were established taking into account the LD_50_ and the dose calculation described in Organisation for Economic Co-operation and Development (OECD) Guideline 407 [21]. Throughout the 30-day treatments, the animals were observed daily for general health and clinical signs of toxicity, whereas body weight changes were recorded on days 0, 7, 14, 21, and 28 of the experiment. At the end of the study period, all animals were fasted overnight before blood sampling. Blood samples were collected from the abdominal aorta under anesthesia with ether in two types of tubes: one with EDTA and the other without additives. The anticoagulated blood (tube with EDTA) was analyzed immediately for hematological parameters. The second tube was centrifuged at 3000 rpm at 4°C for 10 min to obtain the serum for biochemical analysis. Additionally, liver and kidneys were dissected and weighed, and wet sections from these organs were examined histopathologically.

#### 2.5.2. Hematological Parameters

Hematological examination was performed using an automatic hematological analyzer (Abacus 380 Hematology Analyzers). The hematological parameters measured were white blood cells (WBC), red blood cell (RBC), granulocyte (GRA), lymphocyte (LYM), hemoglobin (HGB), hematocrit (HCT), mean corpuscular volume (MCV), mean corpuscular hemoglobin (MCH), mean corpuscular hemoglobin concentration (MCHC), red cell distribution width (RDW), platelets (PLT), and mean platelet volume (MPV).

#### 2.5.3. Serum Biochemistry

Biochemical analysis of serum samples was performed using an automatic chemistry analyzer (COBAS INTEGRA® 400 Plus analyzer.), including albumin (ALB), alkaline phosphatase (ALP), alanine aminotransferase (ALT), aspartate transaminase (AST), bilirubin (BIL), cholesterol (CHOL), triglyceride (TRGL), creatinine (CRE), and urea (URE).

#### 2.5.4. Histopathological Examination of the Liver and Kidney Tissues

Macroscopic examination of vital organs was carried out soon after sacrifice. Liver and kidneys were surgically removed, weighed (absolute organ weight) on an analytical balance, and immediately forwarded to the histopathological processing. The relative organ weight (ROW) of each animal was then calculated as follows: relative organ weight (%) = (organ weight/body weight) × 100 [22]. The fragments of livers and kidneys were fixed in a 10% buffered formalin solution, included in paraffin wax, cut into 3-4 *μ*m sections, and stained with hematoxylin and eosin. The histological sections were then visualized under optical microscopy (Optika Microscopes, Italy) and captured by an Infinity 1 camera microscope with ×40 magnification.

Histopathological analysis consists of the observation of tissue integrity, searching for injuries such as degeneration, necrosis, apoptosis, and infiltration of leukocytes which could indicate signs of toxicity.

### 2.6. Statistical Analysis

Data were expressed as mean ± standard error of the mean (SEM). The differences between groups of the subacute toxicity test were determined by analysis of variance (one-way ANOVA) followed by Dunnett's test. *P* values less than 0.05 were set as the level of significance. The statistical analyses were performed by the GraphPad Prism software version 5.00 for Windows.

## 3. Results

### 3.1. Acute Toxicity of HS Extract in Mice

Table 1 shows the effects of HS aqueous extract in mice after acute oral administration. The signs of toxicity, including anorexia and hypoactivity, were observed at doses of 5000, 7000, and 10000 mg/kg. The mortality rate as well as the acute toxicity of the orally administered HS extract increased progressively as the dose increased from 1000 to 10000 mg/kg. The mortality produced in the animals by the HS extract was used to calculate the LD_50_, which was 5 g/kg.

### 3.2. Subacute Toxicity

#### 3.2.1. Body Weight, Dietary, and Water Intake

**Figure 1:**
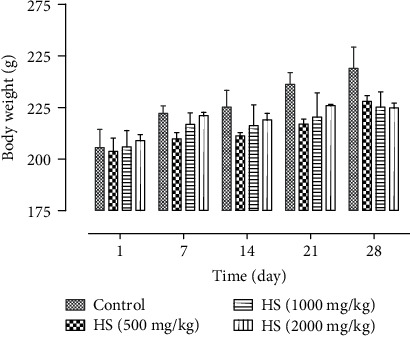
Body weight changes of rats treated with aqueous extract of HS in the 30-day subacute toxicity study. The results are presented as the means ± standard deviation (*n* = 6).

The subacute toxicity of HS extract at all the doses used did not produce any obvious symptoms of toxicity or mortality in all the treated rats. Besides, no significant changes occurred in food and water consumption in rats treated subacutely with repeated oral doses of the aqueous extract (500, 1000, or 2000 mg/kg). Both the control and treated rats appeared healthy at the end and throughout the 30-day period of the study. According to data presented in Figure 1 during the whole experiment period, no significant changes occurred in the weight of treated groups with a dose up to the maximum of 2000 mg/kg compared to the control group (*P* > 0.05). However, the body weight gains of rats treated with 500, 1000, and 2000 mg/kg were all lower than those of the control group, being 11.87%, 9.32%, and 8.59%, respectively, as compared with the control group (18. 67%).

#### 3.2.2. Liver and Kidneys Weights

Absolute and relative organ weights of 30-day treated rats are shown in Table 2. No significant differences in absolute and relative liver and kidney weights were observed between the vehicle control and treatment groups.

#### 3.2.3. Hematological Parameters

The analysis of hematological parameters, which included WBC, LYM, GRA, HCT, MCV, MCH, MCHC, RDW, and MPV in rats treated with HS extract (500, 1000, and 2000 mg/kg) did not differ significantly from those of control rats (Table 3). However, the administration of the HS water extract to rats at 500 mg/kg and 1000 mg/kg resulted in a statistically significant decrease in platelets (PLT) (*P* < 0.05). At the highest dose (2000 mg/kg), the HS extract caused a significant increase in RBC and HGB (*P* < 0.05).

#### 3.2.4. Biochemical Parameters

**Figure 2:**
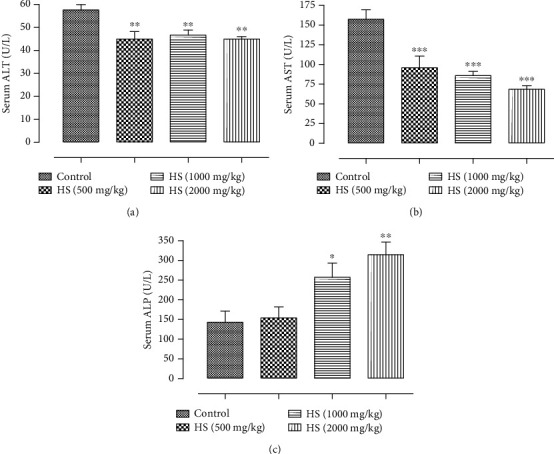
Effect of subacute oral administration of HS extract on (a) ALT (U/L), (b) AST, and (c) ALP (U/L) in serum. Values are presented as the means ± standard deviation. Significant differences were compared with the vehicle control group. ^∗^*P* < 0.05, ^∗∗^*P* < 0.01, and ^∗∗∗^*P* < 0.001. AST: aspartate aminotransferase; ALT: alanine aminotransferase; ALP: alkaline phosphatase.

*(1) Effect of Subacute Treatment of Rats with HS Extract on AST, ALT, and ALP*. The results of the clinical biochemistry parameters assessed in this work, aspartate aminotransferase (AST), alanine aminotransferase (ALT), and alkaline phosphatase (ALP), are summarised in Figure 2. It can be seen that there was a significant increase in ALP observed in groups treated with doses of 1000 mg/kg and 2000 mg/kg compared to the control group (*P* < 0.05; *P* < 0.01). However, the daily oral administration of HS extract to the rats of all groups provoked a significant decrease in the plasma levels of ALT (*P* < 0.01) and AST (*P* < 0.001) compared with the rats of the control group.

**Figure 3:**
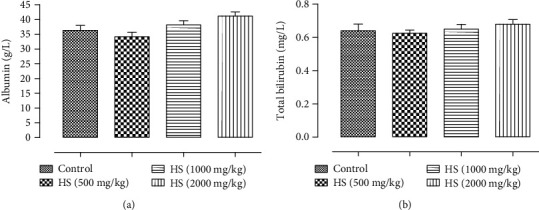
Effect of subacute oral administration of HS extract on (a) albumin and (b) total bilirubin of rats.

*(2) Effect of Subacute Treatments of Rats with HS Extract on Albumin and Total Bilirubin*. The mean values for albumin (A) and total bilirubin (B) of rats after 30 days of intervention have been shown in Figures 3. No significant effects have been observed in the levels of bilirubin and albumin of rats treated with 500, 1000, and 2000 mg/kg of HS extract (*P* > 0.5).

**Figure 4:**
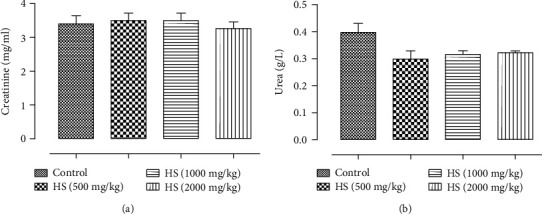
The effect of the oral administration of aqueous extract of HS on (a) creatinine and (b) urea of treated rats. Values are presented as the means ± standard deviation. Significant differences were compared with the vehicle control group. ^∗^*P* < 0.05, and ^∗∗^*P* < 0.01.

*(3) Effect of Subacute Treatment of Rats with HS Extract on Plasma Urea and Creatinine*. After 30 days of treatment, there was no significant alteration in the creatinine and urea levels in the all treated groups (500, 1000, and 2000 mg/kg) compared to control group (Figure 4).

**Figure 5:**
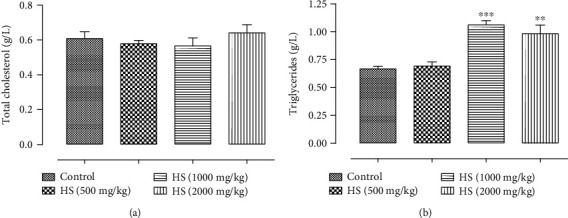
Effect of subacute oral administration of HS extract on plasma total cholesterol (a) and triglycerides (b). Values are presented as the means ± standard deviation. Significant differences were compared with the vehicle control group. ^∗^*P* < 0.05, ^∗∗^*P* < 0.01, and ^∗∗∗^*P* < 0.001.

*(4) Effect of Subacute Treatment of Rats with HS Extract on Plasma Total Cholesterol and Triglycerides*. Plasma concentrations of total cholesterol and triglycerides were investigated in this study to evaluate the effect of HS extract on liver metabolic function (Figure 5). The results indicated that groups treated subacutely with HS at concentrations of 1000 mg/kg and 2000 mg/kg had significantly increased triglycerides levels (*P* < 0.001; *P* < 0.01) compared with the control group. However, the mean values for the cholesterol of rats after 30-day intervention did not significantly differ between control and HS-treated groups.

### 3.3. Histopathological Changes

**Figure 6:**
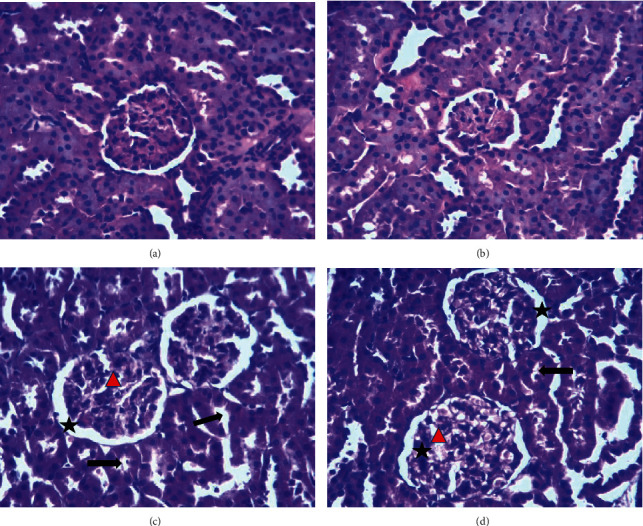
Effect of HS aqueous extract on kidney histology in rats. Histological sections were visualized by staining with hematoxylin and eosin (H and E) and observed by optical microscope (OPTIKA Microscopes, Italy) with magnification ×40. (a) Control rats, (b) rats treated with the aqueous extract of HS (500 mg/kg), (c) rats treated with the aqueous extract of HS (1000 mg/kg), and (d) rats treated with the aqueous extract of HS (2000 mg/kg).

Normal histology of rat kidneys (glomeruli, tubules, interstitium, and blood vessels) was found in the control group (Figure 6(a)), and the group treated subacutely with 500 mg/kg dose of the HS extract (Figure 6(b)). However, the histopathological observations demonstrated that a few minor or slight abnormalities were noted in the histoarchitecture of the kidneys from the groups G3 and G4 (1000 mg/kg and 2000 mg/kg) including a reduction of glomerulus cells (red triangle), a loss of tubular cellular components (black arrow), an expansion of the Bowman space (black star), and distortions in the epithelial membrane of the Bowman capsule (Figures 6(c) and 6(d)).

**Figure 7:**
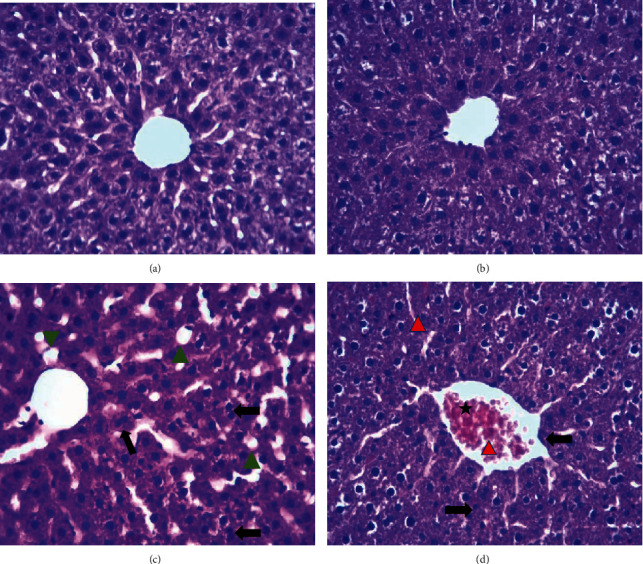
Histopathology of the liver. Light micrographs of the liver sections from different treatment groups. The numbers on the images represent the treatment groups. (a) Control rats, (b) rats treated with the aqueous extract of HS (500 mg/kg), (c) rats treated with the aqueous extract of HS (1000 mg/kg), and (d) rats treated with the aqueous extract of HS (2000 mg/kg).

Histology of the liver sections of control rats showed normal hepatocellular architecture along with well-preserved hepatic cells, and visible central veins and no histologic abnormalities (Figure 7(a)). Rats treated subacutely with 500 mg/kg of HS extract did not cause any adverse effect on the histoarchitecture of hepatocytes (Figure 7(b)). By contrast, the sections of livers taken from HS-treated group (1000 mg/kg) showed some histological changes, such as moderate dilation of sinusoids, mild disorganization of hepatic cords, steatosis, and the hepatocytes while some binucleated cells (Figure 7(c)). Besides, subacute administration of HS extract at the highest dose (2 mg/kg) for 30 days caused a significant alteration in the histoarchitecture of treated rats, showing marked congested dilated central vein, mild disorganization of hepatic cords, and binucleated hepatocytes (Figure 7(d)).

## 4. Discussion

For hundreds of years, natural products, such as medicinal plants, have been the basis for the treatment of various diseases [1]. In screening natural products for the pharmacological activities, assessment, and evaluation of the toxic characteristics of a natural product extract or compound are usually an initial step. Regardless of the pharmacological beneficial effects of HS, detailed knowledge about the poisonous effect of this famous herb is lacking. Hence, the current study was undertaken to evaluate and focus on the acute and subacute toxicity of HS in mice and rats.

The acute toxicity study is utilized to check the harmful effects of an agent to the organism given as a single or short-term exposure [23]. Mainly, the study evaluates the mortality, changes in behavior, body weight, and other spontaneous changes in the overall well-being of the mice. In the present study, the acute toxicity evaluation showed that the oral LD_50_ value of HS extract was 5000 mg/kg. For Hodge and Sterner (2005), with the help of LD_50_ determination, six classes of toxicity are possible: “Class 1=extreme toxicity, LD_50_ < 1 mg/kg; Class 2=high toxicity, LD_50_ at 1–50 mg/kg; Class 3=moderate toxicity, LD_50_ at 50–500 mg/kg; Class 4=low or slight toxicity, LD_50_ at 500–5000 mg/ kg; Class 5=practically nontoxic, LD_50_ at 5000-15,000 mg/kg and Class 6=relatively harmless with LD_50_ > 15,000 mg/kg” (Berezovskaya, 2003). According to this classification and taking account of the LD_50_, we can note that HS with values of LD_50_ = 5000 mg/kg could be graded in the slight toxic category.

The repeated dose toxicity tests provide information on toxic effects, identification of target organs, effects on animal physiology, hematology, the biochemical profile, and histopathology. These tests are required by regulatory agencies to characterize the toxicological potential of any substance [21]. In this study, during subacute exposure, all animals were active and responded positively to stimuli. No deaths and no clinical signs of local or systemic toxic effects were observed. The behavior of the animals was recorded daily (general health and clinical signs of toxicity) and no changes were found [24]. The behavior of all animals in all groups tested was framed as normal for the species.

In general, an increase or decrease in the body weight of an animal has been used as an indicator of an adverse effect of drugs and chemicals [25]. Moreover, the relative organ weight indicates whether the organ has been exposed to injury or otherwise. Impaired organs often have abnormal atrophy [26]. In the present study, the body weight and the relative organ weights of all treated rats did not differ significantly (*P* > 0.05) from those of the control groups. It indicates that the extract did not affect on appetite or adverse effects on the growth of the animals.

Previous studies have shown that hematological parameters were very sensitive and could be used as reliable indicators for determining the intrusion of toxic substances [27]. In our investigation, the treated rats with 2000 mg/kg developed a significant increase of RBC and HB (*P* < 0.05). Therefore, these results are not attributed to any toxicological significance, although these values are within the normal range for the species [28]. Besides, the significant decline of platelet count in rats at a dose of 500 and 1000 mg/kg of HS as compared to normal control group suggested that HS can induce thrombocytopenia, rather produce perturbations in the coagulation cascade. These perturbations are frequently associated with acute liver toxicity and chronic liver disease [29]. The liver is the primary source of many circulating coagulation factors, and acute liver injury and chronic liver disease are each associated with alterations in blood coagulation [30].

Hepatic and renal function are crucial, with one being used for the metabolism of ingestion and the other for excretion of the waste product, respectively [31, 32]. To evaluate the toxicity of any new compound, it is essential to know the state of these two vital organs, which can be verified by biochemical estimation [32]. In this study, liver function and renal function tests were performed. Protein profile and metabolic biomarkers were also measured. Serum levels of three enzymes (ALT, AST, and ALP) are commonly used as clinical biochemistry markers associated with liver damage [33–35]. Among these enzymes, serum levels of ALT and AST of the groups 500, 1000, and 2000 mg/kg/bw were statistically lower when compared to the control. Besides that, all the values found are within the normal range for the species [28]. Therefore, the variations found are not attributed to the clinical significance or the toxic effect of the HS extract. Significant changes in other liver function markers such as ALP were observed in HS extract treatment groups. The subacute exposure of rats to the higher doses (1000 and 2000 mg/kg) of the HS extract produced a significant increase in ALP (*P* < 0.05; *P* < 0.01). Thus, these increase is known to be in response to biliary obstruction [36]. A previous study demonstrates that the elevated serum phosphatase in bile duct obstruction originates in the liver and that its rise in serum is due to the induction of this enzyme within the liver [37]. Moreover, there was a significant increase in triglycerides in rats receiving the HS extract orally at doses of 1000 and 2000 mg/kg, as compared to the control group of rats. An elevation in its level may lead to liver metabolic dysfunction [38]. In contrast, the data showed that there were no significant differences in urea, creatinine, and albumin levels of the groups treated with HS water extract (500 mg/kg, 1000 mg/kg, and 2000 mg/kg) compared to the control. Hence, HS water extract did not alter the kidney function of the rats.

According to the biochemical results, it was reasonable to speculate that the high concentrations of HS extracts may possess toxicity to vital organs in rats. Photomicrographs of the sections of the liver and kidneys of rats treated orally with doses 1000 and 2000 mg/kg of the HS extract for 30 days showed some histological changes, such as moderate dilation of sinusoids, mild disorganization of hepatic cords, binucleated hepatocytes in the liver, slight reduction of glomerulus cells, a loss of tubular cellular components, an expansion of the Bowman space, and distortions in the epithelial membrane of the Bowman capsule in the kidney. Also, the steatosis hepatic was observed only in the treated group with 1000 mg/kg (also proved by biochemical analysis).

Histological assessment of kidneys shows a slight variation in kidneys architecture in rats treated subacutely with HS extract at the doses 1000 and 2000 mg/kg. On the other hand, no significant changes were observed in biochemical markers of renal function in all rats treated. In light of this discrepancy between the biochemical and histological results of the kidneys, it can be said that HS extract does not present nephrotoxicity sufficient to have an alteration of the functions of the kidneys. This may be due to the short period of treatment (30 days), and therefore a chronic study is needed for the complete understanding of the nephrotoxicity of this plant.

Several studies on the constituents of HS showed that this plant is mainly formed of alkaloids (isoquinoline types), glycosides, esters, fatty acids, and other compounds [39]. Two alkaloids (N-methylisosalsoline and carnegine) had been previously described from the aerial parts of *Hammada articulata* ssp. scoparia [16]. A thorough study of this plant material has now led to the isolation of eight minor alkaloids and one flavonoid. The alkaloids include four isoquinolines (isosalsoline, salsolidine, dehydrosalsolidine, and isosalsolidine), one isoquinolone (N-methylcorydaldine), tryptamine, N-omega-methyltryptamine, and one beta-carboline (tetrahydroharman). However, the component(s) of the HS extract which caused toxicity, both in the acute and subacute dose studies, are not known. The acute and subacute toxicities of HS could be explained by the presence in the extract of the alkaloid group. The present study was the first one of its kind that evaluated the short-term subacute toxicity of Haloxylon scoparium aqueous extract; however, it had some limitations. We aimed to assess the possible toxicity effects of HS on major vital organs, such as kidneys and liver, that have more important roles in the detoxification of exogenous chemical substances than other organs; however, in the current study, due to lack of resources, the toxicity of HS was not evaluated in other organs such as nervous, reproductive, and immune systems. Besides, the current study had a shorter duration than typical prechronic toxicity studies recommended by regulatory guidelines. Therefore, it should be taken into account that longer exposure periods (90 days for example) may also yield different results.

## 5. Conclusion

In the present work, the acute and subacute toxicities of the aqueous extract from HS by oral administration in rodents were performed. The result of acute toxicity showed that the oral LD_50_ value of HS extract was lower than 5000 mg/kg, and this extract is regarded as slightly toxic. The findings of 30 days of oral subacute toxicity indicated that HS extract exhibited toxicity to the liver in rats determined by hematological, serum biochemical, and/or histological analyses at high concentrations (1000 mg/kg and 2000 mg/kg).

These results provide valuable preliminary data on the toxic profile of HS. Therefore, further assessments (such as studies of genotoxicity, subchronic toxicity, reproductive toxicity, and compounds toxicity) are required to proceed to clinical studies of this plant.

Finally, it is mandatory to understand that medicinal plants should be investigated and evaluated regarding their toxicities and safeness. So, evaluating the toxicological effects of other medicinal plants currently used by Moroccan people intended remained an important aspect of its assessment for potential toxic effects.

## Figures and Tables

**Table 1:** Mortality and clinical signs of acute toxicity of the aqueous extract of HS administered orally to mice.

Route of administration	Dose of HS extract (g/kg/bw)	Mortality		Toxic symptoms
		D/T	Latency (h)	
Oral	0	0/6	-	None
	1	1/6	<2 h	None
	3	2/6	<2 h	None
	5	3/6	<1 h	Anorexia, hypoactivity
	7	5/6	<1 h	Anorexia, hypoactivity
	10	6/6	<1 h	Anorexia, hypoactivity

**Table 2:** Absolute and relative organ weights of rats treated orally with aqueous extract of HS. Values expressed as mean ± SEM, *n* = 6 animals/group.

Parameters		G1 (control)	G2 (500 mg/kg/bw)	G3 (1000 mg/kg/bw)	G4 (2000 mg/kg/bw)
Absolute organ weights	Liver (g)	8.60 ± 0.63	8.02 ± 0.32	7.83 ± 0.30	7.78 ± 0.42
	Kidney (g)	0.67 ± 0.02	0.62 ± 0.02	0.65 ± 0.03	0.64 ± 0.009

Relative organ weights	BW (g)	244 ± 10.26	228.33 ± 2.68	225.2 ± 7.41	224.8 ± 2.26
	Liver (g)	3.52 ± 0.26	3.51 ± 0.10	3.47 ± 0.13	3.46 ± 0.17
	Kidney (g)	0.27 ± 0.01	0.27 ± 0.01	0.28 ± 0.01	0.28 ± 0.001

**Table 3:** Effect of subacute oral administration of HS extract on hematological parameters of rats. Values are presented as the mean ± standard deviation.

	G1 (control)	G2 (500 mg/kg/bw)	G3 (1000 mg/kg/bw)	G4 (2000 mg/kg/bw)
WBC (10^9^/L)	4.42 ± 0.69	5.10 ± 0.29	3.59 ± 0.21	4.25 ± 0.30
LYM (10^9^/L)	3.75 ± 0.33	3.4 ± 0.37	3.24 ± 0.75	2.61 ± 0.14
GRA (10^9^/L)	0.95 ± 0.10	1.34 ± 0.152	1.08 ± 0.12	1.10 ± 0.04
LYM (%)	61.43 ± 3.87	59.28 ± 1.68	59.84 ± 2.43	59.66 ± 2.04
GRA (%)	28.03 ± 2.18	25.56 ± 2.15	22.13 ± 1.29	27.46 ± 0.63
RBC (10^12^/L)	6.94 ± 0.16	7.34 ± 0.19	7.37 ± 0.22	7.76 ± 0.142^∗^
HGB (g/dL)	12.7 ± 0.19	13.38 ± 0.27	13.44 ± 0.32	13.81 ± 0.18^∗^
HCT (%)	36.81 ± 0.66	38.37 ± 0.92	37.85 ± 1.002	38.37 ± 0.37
MCV (fL)	52.17 ± 1.10	52.16 ± 0.40	51 ± 0.44	51.33 ± 0.42
MCH (pg)	18.25 ± 0.22	18.27 ± 0.23	18.18 ± 0.16	17.36 ± 0.18
MCHC (g/dL)	34.98 ± 0.44	34.92 ± 0.32	35.4 ± 0.28	35.95 ± 0.42
RDW	21 ± 0.51	20.33 ± 0.32	20.16 ± 0.33	20.45 ± 0.29
PLT (10^9^/L)	627.8 ± 20.91	545.6 ± 15.7^∗^	537.7 ± 26.16^∗^	605.5 ± 13.20
MPV (fL)	6.8 ± 0.22	6.46 ± 0.11	6.82 ± 0.12	6.46 ± 0.08

Significant differences were compared with the vehicle control group, ^∗^*P* < 0.05and ^∗∗^*P* < 0.01. WBC: white blood cell count; LYM: lymphocyte; GRA: granulocyte; RBC: red blood cell count; HGB: hemoglobin concentration; HCT: hematocrit; MCV: mean corpuscular volume; MCH: mean corpuscular hemoglobin; MCHC: mean corpuscular hemoglobin concentration; PLT: platelet count; RDW: red blood cell distribution width; MPV: mean platelet volume.

## Data Availability

No data were used to support this study.
